# Supplementary material for: A systematic review and meta-analysis on the effectiveness of an invasive strategy compared to a conservative approach in patients > 65 years old with non-ST elevation acute coronary syndrome
Source: PLoS One. 2020 Feb 27;15(2):e0229491. doi: 10.1371/journal.pone.0229491 (PMC7046207; doi:10.1371/journal.pone.0229491)
Supplement: S1 Checklist — (DOCX) [file pone.0229491.s001.docx]

| **Section/topic** | **#** | **Checklist item** | **Reported on page #** |
| --- | --- | --- | --- |
| **TITLE** | | |  |
| Title | 1 | **A systematic review and meta-analysis on the effectiveness of an invasive strategy compared to a conservative approach in elderly patients with** **non-ST elevation acute coronary syndrome** | **Page 1 (title page), Line 1** |
| **ABSTRACT** | | |  |
| Structured summary | 2 | **Background**: Elderly patients, 65 years old and older, largely represent (>50 %) of hospital-admitted patients with acute coronary syndrome (ACS). Data are conflicting comparing efficacy of early routine invasive (within 48-72 hours of initial evaluation) versus conservative management of ACS in this population. Objective: We aimed to determine the effectiveness of routine early invasive strategy compared to conservative treatment in reducing major adverse cardiovascular events in elderly patients with non-ST elevation (NSTE) ACS. **Data Sources:** We conducted a systematic review of randomized controlled trials through PubMed, Cochrane, and Google Scholar database.  **Study Selection:** The studies included were randomized controlled trials that evaluated the effectiveness of invasive strategy compared to conservative treatment among elderly patients > 65 years old diagnosed with NSTEACS. Studies were included if they assessed any of the following outcomes of death, cardiovascular mortality, myocardial infarction (MI), stroke, recurrent angina, and need for revascularization. Five articles were subsequently included in the meta-analysis.  **Data Extraction:** Three independent reviewers extracted the data of interest from the articles using a standardized data collection form that included study quality indicators. Disparity in assessment was settled by an independent adjudicator.  **Data Synthesis:** All pooled analyses were based on fixed effects model. A total of 2,495 patients were included, 1337 in the invasive strategy group, and 1158 in the conservative treatment group.  **Results:** Meta-analysis showed less incidence of revascularization in the invasive (2%) over conservative treatment groups (8%), with overall risk ratio of 0.31 (95% CI 0.16-0.61, I^2^ =0%). There was also less incidence of stroke in the invasive (2%) versus conservative group (3%) but this was not statistically significant. A significant benefit was noted in the reduction of all-cause mortality (RR 0.63, 95% CI 0.55-0.72, I^2^=84%) and myocardial infarction (RR 0.62, 95% CI 0.49-0.79, I^2^=63%) but with significant heterogeneity.  **Conclusion:** There was a significantly lower rate of revascularization in the invasive strategy group compared to the conservative treatment group. In the reduction of all-cause mortality and MI, there was benefit favoring invasive strategy but with significant heterogeneity. These findings do not support the bias against early routine invasive intervention in the elderly group with NSTEACS. However, further studies focusing on the elderly with larger population sizes are still needed.  **Clinical implications:** These findings do not support the bias against early routine invasive intervention in the elderly group with NSTEACS. This meta-analysis showed that an early invasive strategy appears to be beneficial in suitable elderly patients > 65 years old with NSTEACS.  **Limitations**: Although an early invasive strategy may be favorable among elderly patients presenting with NSTEACS, the certainty of benefit versus risk still needs to be supported by larger clinical trials and registries with uniform age cutoff for elderly, particularly > 65 years old, to provide high generalizability and statistical power. Current risk scoring systems such as the GRACE (Global Registry of Acute Coronary Events) Score, TIMI (Thrombolysis in Myocardial Infarction) Risk Score, and CRUSADE Bleeding Score are recommended in the initial evaluation of elderly patients presenting with NSTEACS. A special risk scoring may be developed to more accurately identify those who are suitable for an early invasive strategy, with an expected larger outcome and survival benefit.  (We do not have a systematic review registration number) | **Page 2-3, Lines 27-57**  **Page 19, Lines 306-316**  **Page 19, Lines 306-316** |
| **INTRODUCTION** | | |  |
| Rationale | 3 | Despite the guidelines, older patients are less likely to undergo procedures after an NSTEACS than younger patients due in part to patient and practitioner concerns about the increased risk of complications.^7,8,9^  Due to conflicting results of studies, lack of specific recommendations from the above-mentioned guidelines, and the paucity of data on early invasive strategy versus conservative treatment for NSTEACS in elderly patients, this meta-analysis was conducted to focus on this special population to compare benefits and risks of early invasive therapy versus conservative management. | **Page 4, Lines 81-89** |
| Objectives | 4 | ***Objectives***  ***General:*** To determine the effectiveness of invasive strategy compared to conservative treatment in reducing MACE among elderly patients with NSTEACS.  ***Specific:***  Among elderly patients with NSTEACS, to determine the effectiveness of invasive strategy compared to conservative treatment, in 6 months (short-term) to 3 years (long-term), in reducing:   1. Death or all-cause mortality; 2. Cardiovascular mortality; 3. Myocardial infarction (MI); 4. Stroke; 5. Recurrent angina 6. Need for revascularization. | **Page 4-5, Lines 96-109** |
| **METHODS** | | |  |
| Protocol and registration | 5 | Prior to the conduct of the research, the study was registered and approved by the Committee on Research of Manila Doctors Hospital. | **Page 5, Lines 112-114** |
| Eligibility criteria | 6 | ***a. Study Characteristics***  ***Population:* Page 5, Line 117-118, under “Criteria for considering studies for this review under Methodology”:**  “…among elderly patients > 65 years old diagnosed with NSTEACS…”  ***Intervention:* Page 5, Line 116-117, under “Criteria for considering studies for this review under Methodology”:**  “Randomized controlled trials that evaluated the effectiveness of invasive strategy compared to conservative treatment…”; “Invasive strategy” is considered the intervention.  ***Comparator:* Page 5, Line 116-117, under “Criteria for considering studies for this review under Methodology”:**  “Randomized controlled trials that evaluated the effectiveness of invasive strategy compared to conservative treatment…”; “Conservative treatment” is considered the comparator.  ***Outcomes:* Page 5, Line 118-119, under “Criteria for considering studies for this review under Methodology”:**  “Studies were included if any of the outcomes assessed were: death, cardiovascular mortality, MI, stroke, recurrent angina, and need for revascularization.”  ***Study Designs:* Page 5, Line 116, under “Criteria for considering studies for this review under Methodology”:**  **“**The studies included were randomized controlled trials…”  ***b. Report Characteristics***  All studies included were published data, all in English language. | **Page 5, Lines 116-119** |
| Information sources | 7 | Systematic computerized search was performed using the Pubmed and Cochrane databases. The last search was run on 10 August 2017.  ***Contact with study authors:*** One article was possibly eligible but did not report the event rates per treatment group. To access needed data in this particular study, correspondence with the author via email was done, but with no reply from the author. | **Page 6, Lines 136-137; 141-142**  **Page 6, under “Search methods for identification of studies” Lines 149-152:** |
| Search | 8 | Systematic computerized search (APPENDIX A) was performed using the Pubmed and Cochrane databases. MESH and free text of the following main key terms were used: “randomized controlled trials”, “elderly”, “non-ST elevation acute coronary syndrome”, “invasive strategy”, “conservative management”, “invasive strategy versus conservative strategy”, “major adverse cardiovascular events”, “all-cause mortality”, “cardiovascular mortality”, “myocardial infarction”, “stroke”, “recurrent angina”, “need for revascularization”. | **Page 6, Lines 136-141**  ***In appendix:* Page 22-25, Lines 386-387 (Appendix A)** |
| Study selection | 9 | Eligibility assessment was performed independently in an unblinded standard manner by three reviewers. The literature search identified 322 possible articles. Of these, 69 were relevant, particularly they involved studies related to ACS. Prospective cohort studies and post hoc analyses were excluded. Of the 69 articles, 55 were excluded due to different intervention since they did not involve comparing invasive versus conservative management in ACS. After assessing 14 articles for eligibility, 8 articles with different population and methods were excluded (details for reasons for exclusion are listed in APPENDIX D). One article was possibly eligible but did not report the event rates per treatment group. To access needed data in this particular study, correspondence with the author via email was done, but with no reply from the author until the time of writing. Five articles were subsequently included in the meta-analysis (Figure 1). | **Page 6-7, Lines 143-153**  **Page 7, Figure 1, Lines 154-155** |
| Data collection process | 10 | Three independent reviewers extracted the data of interests using a standardized data collection form and individually appraised each trial. The reviewers discussed the quality of included trials, outcome to be collected, and risk of biases. Disparity in assessment was settled by an independent adjudicator. | **Page 7, Lines 158-161**  **Appendix B, Page 26-30, Line 394-408, shows the format of the data extraction tool** |
| Data items | 11 | Five randomized controlled trials involving a total of 2,495 patients met the inclusion criteria. The data on population characteristics, intervention type, and measured outcomes were extracted from each trial (Table 1) | **Page 8, Lines 174-176**  **Study details in Table 1,** **Page 8-12** |
| Risk of bias in individual studies | 12 | ***Assessment of risk bias of included trials:***  Three independent reviewers extracted the data of interest using a standardized data collection form and individually appraised each trial. The reviewers discussed the quality of included trials, outcomes to be collected, and risks of bias. Disparity in assessment was settled by an independent adjudicator. The assessment of random sequence generation, allocation concealment, incomplete outcome data, blinding of participants and personnel, blinding of outcome assessment, and intention-to-treat analysis was done using the quality scale for meta-analytic review, the Cochrane Collaboration Tool for Risk of Bias.  The Cochrane collaboration tool was used to assess the risk of bias. The random sequence generation, allocation concealment, incomplete outcome data, blinding of participants and personnel, blinding of outcome assessment, and intention-to-treat analysis were evaluated for each trial. All included trials were assessed to have low risk for bias (Table 2). | **Page 7-8, Lines 157-164**  .  **Page 13, Lines 191-194**  **Table 2, Page 13-14, Lines 196-197** |
| Summary measures | 13 | Review Manager 5.3 was used to analyze the data. Analysis of dichotomous data was done using risk ratio, 95% confidence interval, and Mantel-Haenszel method with fixed effects model. Heterogeneity between trials was tested using a standard Chi-square test and I^2^ statistics. The p-value of <0.10 was considered to be statistically significant and I^2^ of ≥50% is considered to have high heterogeneity. | **Page 7, Lines 167-171** |
| Synthesis of results | 14 | Heterogeneity between trials was tested using a standard Chi-square test and I^2^ statistics. The p-value of <0.10 was considered to be statistically significant and I^2^ of ≥50% is considered to have high heterogeneity. | **Page 7, Lines 169-171** |

Page 1 of 2

| **Section/topic** | **#** | **Checklist item** | **Reported on page #** |
| --- | --- | --- | --- |
| Risk of bias across studies | 15 | Kindly refer to **Table 2. Quality assessment table** | **Table 2, Page 13-14, Lines 196-197** |
| Additional analyses | 16 | Analysis of data was done using the fixed effects model. An attempt to analyze data using the random effects model was done but analysis showed similar results. |  |
| **RESULTS** | | |  |
| Study selection | 17 | Kindly refer to **Figure 1: Search strategy for identification of studies** | **Figure 1, Page 7, Lines 153-155** |
| Study characteristics | 18 | Kindly refer to **Table 1. Characteristics of included trials** | **Table 1, Page 8-12, Lines 181-182** |
| Risk of bias within studies | 19 | Kindly refer to **Table 2. Quality assessment table**. | **Table 2, Page 13-14, Lines 196-197** |
| Results of individual studies | 20 | **Effects of intervention on outcomes of interest**   1. ***All-cause mortality***   A total of 242 among 1338 (18 %) elderly patients with NSTEACS died in the Invasive Strategy Group; while 296 died among 1158 (26 %) patients in the Conservative Group (Figure 2). The pooled analysis of all-cause mortality showed statistically significant benefit of invasive over conservative strategy with an overall risk ratio of 0.63 (95% CI 0.55 to 0.72) but with significant heterogeneity (p value of 0.0001, I^2^ =84%).  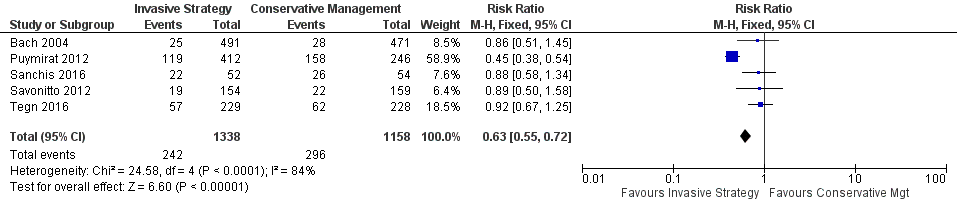  **Figure 2. Comparison between invasive and conservative strategy with the outcome of all-cause mortality**   1. ***Myocardial infarction***   In the Invasive Strategy Group, there were 89 events of MI among a total of 926 (10 %) patients; while there were 142 among 912 (16 %) patients in the Conservative Group (Figure 3). The pooled analysis showed that invasive strategy is beneficial over conservative treatment in preventing MI with an overall risk ratio of 0.62 (95% CI 0.49 to 0.79) but with significant heterogeneity (p value of 0.0001, I^2^ = 63%).  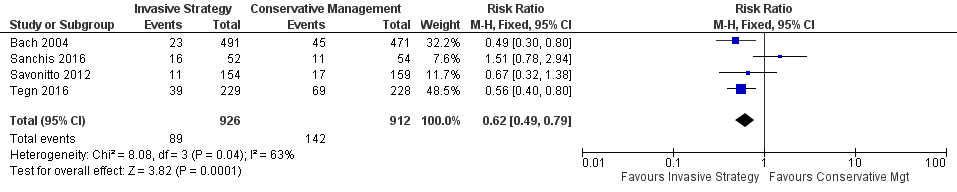  **Figure 3. Comparison between invasive and conservative strategy with the outcome of myocardial infarction**   1. ***Stroke***   Among the five trials, Savonitto et al. (2012), Tegn (2016), and Bach (2004) reported the outcomes of stroke (Figure 4). In the Invasive Strategy Group, there were 13 events of stroke among 874 (2%) patients; while there were 24 among 858 (3%) patients in the Conservative Group. The pooled analysis showed that early invasive strategy was favored over conservative treatment in preventing stroke but no statistically significant benefit with overall risk ratio of 0.53 (95% CI 0.27-1.03, I^2^ =0%).  **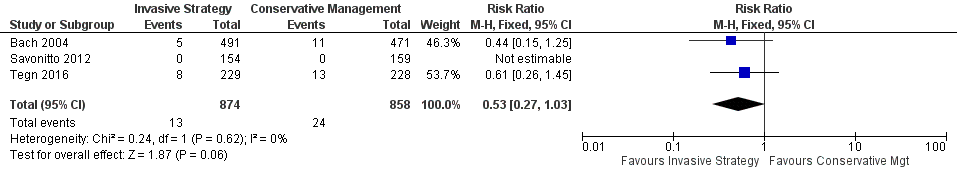**  **Figure 4. Comparison between invasive and conservative strategy with the outcome of stroke**   1. ***Need for revascularization***   In elderly patients with NSTEACS, there were a total of 10 patients among 435 (2%) who needed revascularization in the Invasive Group while there were 34 patients among 441 (8%) in the Conservative Group (Figure 5). The pooled analysis for need for revascularization showed statistically significant benefit with an overall risk ratio of 0.31 (95% CI 0.16 to 0.61) with no significant heterogeneity (p value of 0.0006, I^2^ =0%).  **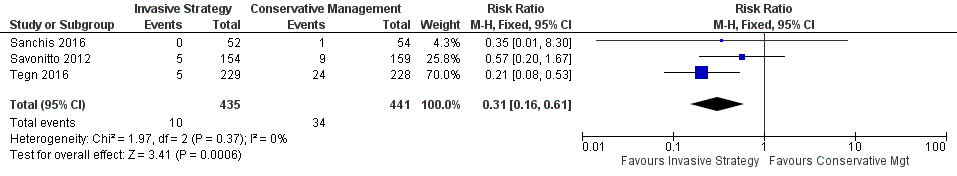**  **Figure 5. Comparison between invasive and conservative strategy with the outcome of need for revascularization**   1. ***Outcomes for cardiovascular mortality and recurrent angina***   Among the five trials, only one trial assessed the outcomes of cardiovascular mortality and recurrent angina.^12^ The cardiovascular mortality incidence in the invasive versus the control group was 10% and 11 %, respectively, showing a non-statistical benefit of invasive over conservative treatment (RR 0.87, 95% CI, 0.49-1.56, p=0.65). Likewise, an invasive strategy showed a non-statistical benefit over conservative treatment in reducing recurrent angina (RR 0.81, 95% CI 0.45–1.46, p=0.49). | **Page 14-17, Lines 200-250** |
| Synthesis of results | 21 | Meta-analysis of data from the five trials included in this study showed that an early invasive strategy appears to be beneficial in suitable elderly patients > 65 years old with NSTEACS. There was significantly less need for revascularization in the invasive strategy group compared to the conservative treatment group. This finding implies that more patients in the conservative group clinically worsened during their course in the ward, requiring revascularization. It is also possible that early anatomic definition of the diseased coronaries may help the attending physician optimize an appropriate evidence-based management of the patient. The studies that evaluated the outcomes of revascularization stated that the indications for revascularization in the conservative group were: positive pre-discharge stress test, poor in-hospital outcomes, recurrent ischemia, reinfarction, malignant ventricular arrhythmias, refractory angina, and heart failure.^12-14^ Some patients who subsequently required revascularization could have probably been better off with an early invasive approach.  For the outcomes of death and MI, an invasive strategy showed a statistically significant benefit over conservative treatment but with significant heterogeneity. The possible sources of heterogeneity for the outcomes of death and MI may be the small number of events and sample sizes. In two studies, the elderly population was just a subgroup analysis of the total population.^10-11^ Hence, the population in the subgroup analysis may not be powered enough to detect the differences in the intervention and outcomes of interest. Furthermore, there were differences in age cutoffs and follow-up period. Two studies had age cutoffs of 75 years^11,13^ while the other three studies had age cutoffs of 65, 70, and 80 years.^10,12,14^ Possible clinical differences in outcomes may exist in these age brackets of the elderly population. In terms of follow-up periods, two studies had follow-up of 3 years^11,12^; one had follow-up period of 3 months to 3 years^14^; one had follow-up of 1 year^13^; while one had follow-up of 6 months and 1 year^10^. However, despite the heterogeneity, data from these studies clustered on the direction towards benefit favoring invasive over conservative strategy.  In the reduction of stroke, invasive strategy showed benefit over conservative treatment but this was not statistically significant. The outcomes for cardiovascular mortality and recurrent angina were assessed only in one study, which showed also a non-statistically significant benefit of invasive strategy over conservative treatment among elderly NSTEACS patients. | **Page 17-18, Lines 253-281** |
| Risk of bias across studies | 22 | Kindly refer to **Table 2. Quality assessment table** | **Table 2, Page 13-14, Lines 196-197** |
| Additional analysis | 23 | Give results of additional analyses, if done (e.g., sensitivity or subgroup analyses, meta-regression | **See Item 16.** |
| **DISCUSSION** | | |  |
| Summary of evidence | 24 | Meta-analysis of data from the five trials included in this study showed that an early invasive strategy appears to be beneficial in suitable elderly patients > 65 years old with NSTEACS. There was significantly less need for revascularization in the invasive strategy group compared to the conservative treatment group. This finding implies that more patients in the conservative group clinically worsened during their course in the ward, requiring revascularization. It is also possible that early anatomic definition of the diseased coronaries may help the attending physician optimize an appropriate evidence-based management of the patient. The studies that evaluated the outcomes of revascularization stated that the indications for revascularization in the conservative group were: positive pre-discharge stress test, poor in-hospital outcomes, recurrent ischemia, reinfarction, malignant ventricular arrhythmias, refractory angina, and heart failure.^12-14^ Some patients who subsequently required revascularization could have probably been better off with an early invasive approach.  For the outcomes of death and MI, an invasive strategy showed a statistically significant benefit over conservative treatment but with significant heterogeneity. The possible sources of heterogeneity for the outcomes of death and MI may be the small number of events and sample sizes. In two studies, the elderly population was just a subgroup analysis of the total population.^10-11^ Hence, the population in the subgroup analysis may not be powered enough to detect the differences in the intervention and outcomes of interest. Furthermore, there were differences in age cutoffs and follow-up period. Two studies had age cutoffs of 75 years^11,13^ while the other three studies had age cutoffs of 65, 70, and 80 years.^10,12,14^ Possible clinical differences in outcomes may exist in these age brackets of the elderly population. In terms of follow-up periods, two studies had follow-up of 3 years^11,12^; one had follow-up period of 3 months to 3 years^14^; one had follow-up of 1 year^13^; while one had follow-up of 6 months and 1 year^10^. However, despite the heterogeneity, data from these studies clustered on the direction towards benefit favoring invasive over conservative strategy.  In the reduction of stroke, invasive strategy showed benefit over conservative treatment but this was not statistically significant. The outcomes for cardiovascular mortality and recurrent angina were assessed only in one study, which showed also a non-statistically significant benefit of invasive strategy over conservative treatment among elderly NSTEACS patients.  Overall, this study does not support the relatively conservative tendency when dealing with elderly patients with NSTEACS in real-life clinical setting. The elderly population is considered a high-risk group wherein more than half the mortality in NSTEACS occur^5^ and a more aggressive approach in suitable patients may be more appropriate and beneficial. Among people who die of ischemic heart disease, 83% were >65 years of age.^1^ This mortality rate is expected to increase in the forthcoming decades due to improving life expectancy of the elderly. Age is one of the most important predictors of risk in NSTEACS. Each 10-year increase in age results in a 75% increase in hospital mortality in ACS patients.^15^ Despite the relatively higher risk in this age group, elderly ACS patients are under-represented in clinical trials such that subjects older than 75 years of age account for less than 10%, and those older than 85 years account for less than 2% of all NSTEACS subjects. ^7^ This highlights the need for more clinical trials and studies in this age group.  Data from the CRUSADE (Can Rapid Risk Stratification of Unstable Angina Patients Suppress Adverse Outcomes with Early Implementation of the American College of Cardiology/American Heart Association Guidelines) registry showed that NSTEMI patients aged ≥ 65 years who experienced an in-hospital major bleed had a 33% increased risk of 30-day mortality.^16^ However, the advancement of equipment and technique has made PCI safer for even very elderly patients (≥ 90 years of age) with high success rates and declining major bleeding risk.^17^ | **Page 17-18, Lines 253-298** |
| Limitations | 25 | The possible sources of heterogeneity for the outcomes of death and MI may be the small number of events and sample sizes. In two studies, the elderly population was just a subgroup analysis of the total population.^10-11^ Hence, the population in the subgroup analysis may not be powered enough to detect the differences in the intervention and outcomes of interest. Furthermore, there were differences in age cutoffs and follow-up period. Two studies had age cutoffs of 75 years^11,13^ while the other three studies had age cutoffs of 65, 70, and 80 years.^10,12,14^ Possible clinical differences in outcomes may exist in these age brackets of the elderly population. In terms of follow-up periods, two studies had follow-up of 3 years^11,12^; one had follow-up period of 3 months to 3 years^14^; one had follow-up of 1 year^13^; while one had follow-up of 6 months and 1 year^10^. However, despite the heterogeneity, data from these studies clustered on the direction towards benefit favoring invasive over conservative strategy. | **Page 17-18, Lines 266-277** |
| Conclusions | 26 | Results of this meta-analysis suggest some benefits with an early invasive strategy compared to a conservative treatment approach in the management of elderly patients with NSTEACS. There was a significantly lower rate of revascularization in the invasive strategy group compared to the conservative treatment group. A statistically significant benefit favoring invasive strategy was also noted in the reduction of death and myocardial infarction but with significant heterogeneity. These findings do not support the bias against early routine invasive intervention in the elderly group with NSTEACS.  Although an early invasive strategy may be favorable among elderly patients presenting with NSTEACS, the certainty of benefit versus risk still needs to be supported by larger clinical trials and registries with uniform age cutoff for elderly, particularly > 65 years old, to provide high generalizability and statistical power. | **Page 19, Lines 301-311:** |
| **FUNDING** | | |  |
| Funding | 27 | This study was investigator-initiated, and there was no funding source. |  |

*From:*  Moher D, Liberati A, Tetzlaff J, Altman DG, The PRISMA Group (2009). Preferred Reporting Items for Systematic Reviews and Meta-Analyses: The PRISMA Statement. PLoS Med 6(7): e1000097. doi:10.1371/journal.pmed1000097
