# Supplementary material for: A systematic review and meta-analysis on the effectiveness of an invasive strategy compared to a conservative approach in patients > 65 years old with non-ST elevation acute coronary syndrome
Source: PLoS One. 2020 Feb 27;15(2):e0229491. doi: 10.1371/journal.pone.0229491 (PMC7046207; doi:10.1371/journal.pone.0229491)
Supplement: S3 Appendix — (DOCX) [file pone.0229491.s004.docx]

## S3 Appendix. Sample data extraction template.

| Trial ID | Extractor | Year of publication |
| --- | --- | --- |
| Title | | |
| Authors | | |
| Citation | | |

# Participants

| **Inclusion criteria:**  **Exclusion criteria:** |
| --- |

# Intervention

| **Treatment group:**  **Control/Comparison group:** |
| --- |

**Method**

|  |
| --- |

# Quality assessment/ risk of bias table

| Domain | Judgement  Low Risk/ High Risk/ Unclear | Support for Judgement/  Description |
| --- | --- | --- |
| Method of Random sequence  Generation (Selection Bias) |  |  |
| Method of allocation  Concealment (Selection Bias) |  |  |
| Incomplete Outcome Data/Loss of participants to follow up (Attrition Bias) |  |  |
| Blinding of Participants and Personnel (Performance Bias) |  |  |
| Blinding of Outcome Assessment (Detection Bias) |  |  |
| Selective Reporting/ Intention to treat analysis (Reporting Bias) |  |  |
| Other Bias |  |  |

**Outcomes**

|  | Outcome Measures (Dichotomous) | Total = | | | |
| --- | --- | --- | --- | --- | --- |
|  |  | Intervention group  n = | | Control groupn = | |
|  |  | Events | total | events | Total |
|  | Primary: |  |  |  |  |
| 1 |  |  |  |  |  |
|  | Secondary: |  |  |  |  |
| 2 |  |  |  |  |  |
| 3 |  |  |  |  |  |
