# Supplementary material for: A systematic review and meta-analysis on the effectiveness of an invasive strategy compared to a conservative approach in patients > 65 years old with non-ST elevation acute coronary syndrome
Source: PLoS One. 2020 Feb 27;15(2):e0229491. doi: 10.1371/journal.pone.0229491 (PMC7046207; doi:10.1371/journal.pone.0229491)
Supplement: S4 Appendix — (DOCX) [file pone.0229491.s005.docx]

## S4 Appendix. Summary of results of the six included randomized controlled trials.

**Tegn et al., 2016. After Eighty Study**

Invasive versus conservative strategy in patients aged 80 years or older with non-ST-elevation myocardial infarction or unstable angina pectoris (After Eighty study): an open-label randomised controlled trial

|  | Outcome Measures (Dichotomous) | Total = 457 | | | |
| --- | --- | --- | --- | --- | --- |
|  |  | Intervention group  n = 229 | | Control groupn = 228 | |
|  |  | Events | Total | Events | Total |
| 1 | All-Cause Mortality | 57 | - | 62 | - |
| 2 | Cardiovascular Mortality | Not reported |  | Not reported |  |
| 3 | Myocardial infarction | 39 | - | 69 | - |
| 4 | Stroke | 8 | - | 13 | - |
| 5 | Recurrent angina | Not reported | - | Not reported | - |
| 6 | Need for revascularization | 5 | - | 24 | - |

**Sanchis et al., 2016.**

Randomized comparison between the invasive and conservative strategies in comorbid

elderly patients with non-ST elevation myocardial infarction

|  | Outcome Measures (Dichotomous) | Total = 106 | | | |
| --- | --- | --- | --- | --- | --- |
|  |  | Intervention group  n = 52 | | Control groupn = 54 | |
|  |  | Events | Total | Events | Total |
| 1 | All-Cause Mortality | 22 | - | 26 | - |
| 2 | Cardiovascular Mortality | Not reported | - | Not reported |  |
| 3 | Myocardial infarction | 16 | - | 11 | - |
| 4 | Stroke | Not reported | - | Not reported | - |
| 5 | Recurrent angina | Not reported | - | Not reported | - |
| 6 | Need for revascularization | 0 | - | 1 | - |

**Wallentin et al., 2016. FRISC-II**

Early invasive versus non-invasive treatment in patients with non-ST-elevation acute coronary syndrome (FRISC-II): 15 year follow-up of a prospective, randomised, multicentre study

|  | Outcome Measures (Dichotomous) | Total N= 1,292 elderly or > 65 age subgroup (among a total population of 2,457 initially recruited patients) | | | |
| --- | --- | --- | --- | --- | --- |
|  |  | Intervention group  n = 656 | | Control groupn = 636 | |
|  |  | Events | Total | Events | Total |
| 1 | All-Cause Mortality | 365 | n=648  with > 65 age subgroup  (2,421 among 2,457 have known survival status) | 350 | n=624  with > 65 age subgroup  (2,421 among 2,457 have known survival status) |
| 2 | Cardiovascular Mortality | 140 | n=648  with > 65 age subgroup  (2,421 among 2,457 have known survival status) | 153 | n=624  with > 65 age subgroup  (2,421 among 2,457 have known survival status) |
| 3 | Myocardial infarction | Not included since the outcome was composite and death and myocardial infarction | - | - | - |
| 4 | Stroke | Not reported | - | Not reported | - |
| 5 | Recurrent angina | Not reported | - | Not reported | - |
| 6 | Need for revascularization | Not reported | - | Not reported | - |

**Savonitto et al., 2012. Italian Elderly ACS Study**

Early Aggressive Versus Initially Conservative Treatment in Elderly Patients With Non–ST-Segment Elevation Acute Coronary Syndrome

|  | Outcome Measures (Dichotomous) | Total = 313 | | | |
| --- | --- | --- | --- | --- | --- |
|  |  | Intervention group  n = 154 | | Control groupn = 159 | |
|  |  | events | Total | Events | Total |
| 1 | All-Cause Mortality | 19 |  | 22 |  |
| 2 | Cardiovascular Mortality | 16 |  | 17 |  |
| 3 | Myocardial infarction | 11 |  | 17 |  |
| 4 | Stroke | 0 |  | 0 |  |
| 5 | Recurrent angina | 0 |  | 4 |  |
| 6 | Need for revascularization | 5 |  | 9 |  |

**Puymirat et al., 2012. FAST-MI**

Use of Invasive Strategy in Non–ST-Segment Elevation Myocardial Infarction Is a Major Determinant of Improved Long-Term Survival

FAST-MI (French Registry of Acute Coronary Syndrome)

|  | Outcome Measures (Dichotomous)  In the Subgroup > 75 years old | Total = 658 | | | |
| --- | --- | --- | --- | --- | --- |
|  |  | Intervention group  n = 412 | | Control groupn = 246 | |
|  |  | Events | Total | Events | Total |
| 1 | All-Cause Mortality | 119 | - | 158 | - |
| 2 | Cardiovascular Mortality | Not reported | - | Not reported |  |
| 3 | Myocardial infarction | Not reported | - | Not reported | - |
| 4 | Stroke | Not reported | - | Not reported | - |
| 5 | Recurrent angina | Not reported | - | Not reported | - |
| 6 | Need for revascularization | Not reported | - | Not reported | - |

**Bach et al., 2004.**

The Effect of Routine, Early Invasive Management on Outcome for Elderly Patients with Non–ST Segment Elevation Acute Coronary Syndromes

|  | Outcome Measures (Dichotomous) at 6 Months | Total = 962 | | | |
| --- | --- | --- | --- | --- | --- |
|  |  | Intervention group  n = 491 | | Control groupn = 471 | |
|  |  | Events | Total | Events | Total |
| 1 | All-Cause Mortality | 5.3 % (25) | - | 5.9% (28) | - |
| 2 | Cardiovascular Mortality | Not reported | - | Not reported |  |
| 3 | Myocardial infarction | 4.7 % (23) | - | 9.6 % (45) | - |
| 4 | Stroke | 1.02 % (5) | - | 2.34 %11 | - |
| 5 | Recurrent angina | Not reported | - | Not reported | - |
| 6 | Need for revascularization | Not reported | - | Not reported | - |
