# Supplementary material for: A systematic review and meta-analysis on the effectiveness of an invasive strategy compared to a conservative approach in patients > 65 years old with non-ST elevation acute coronary syndrome
Source: PLoS One. 2020 Feb 27;15(2):e0229491. doi: 10.1371/journal.pone.0229491 (PMC7046207; doi:10.1371/journal.pone.0229491)
Supplement: Attachment — Submitted filename: Response to Reviewers.docx [file pone.0229491.s006.docx]

January 2, 2019

Carmine Pizzi

Academic Editor

PLOS ONE

Dear Ms. Pizzi:

Thank you for all the comments of the reviewers of our manuscript with the following code and title: PONE-D-19-21413; “A systematic review and meta-analysis on the effectiveness of an early invasive strategy compared to a conservative approach in elderly patients with non-ST elevation acute coronary syndrome”.

The following is our response to each point raised by the reviewers:

**I. RE: JOURNAL REQUIREMENTS**

A. In the Declaration of Conflict of Interest in page 31, we have maintained the statement: "I have read the journal's policy and the authors of this manuscript have the following competing interests: RRC is a member of advisory board or speakers’ pool of Servier, Boehringer Ingelheim, Menarini, LRI-Therapharma, Sanofi, UAP Pharma, Unilab; MTR is a member of speakers’ pool of Novartis, Servier, Astra Zeneca; the rest declare no conflict of interest."

We have added and confirmed that this does not alter our adherence to all PLOS ONE policies on sharing data and materials, by including the following statement: "This does not alter our adherence to  PLOS ONE policies on sharing data and materials.”

B. We have revised the location of “Figures 7-11” from Appendix to within the text of Methodology, under “Description of Studies”, in pages 18-20. They were now labeled as Figures 2-6”.

**II. RE: REVIEW COMMENTS TO THE AUTHOR**

A. **Comments of Reviewer #1:**

*Reviewer #1: The paper is very interesting.
Major issues.
1. Elderly patients in the title and abstract and the whole paper should be better defined>older than 65 years old is not "elderly". Maybe adding median age should be better
2. Abstract>in the methods authors state that they used fixed effect, although with high inconstency (i2 90%). Fixed effect should not be used for levels of I2 more than 30%. Please follow cochrane guidelines.
3. Frailty should be largely discusse, as may deeply impact on management of these patients (quote PMID: 28143778 ).
4. Lenght of dapt in these patients remains an issue, and should
a) explored with meta. regression analysis
b) discussed (quote on PMID: 30862233)*

**Our response to comments of Reviewer #1:**

1. We have already revised the terms “elderly patients” to “patients ≥ 65 years old”. The mean ± SD or median age of the population reported per trial were added to the characteristics of included studies in Table 1 (pages 9-16).
2. Following Cochrane Guidelines, the analysis was revised from Fixed Effects Model to Random Effects for the pooled analysis of studies with significant heterogeneity.
3. Frailty was now included in the Discussion part on pages 28-29.
4. The main objective of the study was to compare outcomes between routine invasive and conservative or noninvasive strategy. So the use of either single antiplatelet therapy (SAPT) or double antiplatelet therapy (DAPT)—though still an important unresolved issue in this age group in real-world practice— was not within the scope of the study. Furthermore, current available data suggest that in elderly and very elderly patients who were conservatively managed after AMI, the protective effects of SAPT and DAPT were comparable. (Lin et al. BMC Geriatrics 2018; 18:86). For our study, included now in the discussion on page 30 is the citation of findings from the cohort study of the RENAMI registry concerning the duration of DAPT. Mentioned in the paragraph is how the finding from this study could help us in decision-making regarding the duration of DAPT for older patients who underwent PCI under the invasive strategy.
B. **Comments of Reviewer #2:**

*Reviewer #2: This metanalysis would basically be helpful as it tries to clarify a relevant point about management of NSTE-ACS elderly patients.
The following points limit, however, the conclusions deriving from the analysis.
1. Some more information would be helpful to put the results in the right perspective. Thus, the authors should: a) report the average duration of follow-up for each of the end-points; b) to specify the proportion of patients undergoing by-pass surgery vs. percutaneous coronary intervention; c) the proportion of patients who underwent coronary revascularization during hospital staying in the conservative group.*

*2. Figure 2 shows that the study by Puymirat et al. achieved results strongly different from the other studies as far as global mortality is concerned. Accordingly, the authors should at least present the results of the metanalysis also after removing this study.
3. Although significantly higher than that of the interventional group, coronary revascularization in the conservative group was rather low (8% vs. 2%), which may not justify a broad application of the invasive strategy to all elderly patients.*

**Our response to comments of Reviewer #2:**

1. a. The average duration of follow-up for each endpoint was now included in the Results part on pages 23-27

b. and c. The proportion of patients undergoing by-pass surgery vs. percutaneous coronary intervention and the proportion of patients who underwent coronary revascularization during hospital stay in the conservative group were now included in the “Description of Studies” under Methodology on pages 10-18.

2. The study by Puymirat et al. was still included in the analysis of the outcome of all-cause mortality. Using the random effects model (due to the significant heterogeneity), the revised analysis showed non-significant results for this particular outcome. Since only the study by Puymirat et al. was largely different from the other studies in the outcome of all-cause mortality, a pooled analysis that excluded this study was done (illustrated in Appendix E). The results showed that invasive strategy still had no significant effect versus conservative strategy on the outcome of all-cause mortality.
3. Although the rate of coronary revascularization in the conservative group was still relatively low (8%), the considerably lower rate of coronary revascularization in the routinely invasive group (2%) offers a tenable argument against the prevailing real-world bias of a preferred conservative approach even in non-frail senior patients ≥ 65 years old. Although decision on management strategy still needs to be done on a case-to-case basis with frailty as a major consideration, the study’s findings nonetheless offers a clinically useful insight that justifies a more aggressive approach in this age group if clinically warranted.

We hope that we have addressed all feedback pointed out by the reviewers. We are looking forward to your most favorable response to this revised manuscript.

Thank you very much in behalf of our research group.

Respectfully,

Joan Dymphna P. Reaño, MD

Cardiology Fellow

Manila Doctors Hospital
